# Cryptogenic ischemic stroke in cardiac transthyretin amyloidosis and sinus rhythm: a case report

**DOI:** 10.3389/fcvm.2024.1386733

**Published:** 2024-05-13

**Authors:** Angela Napolitano, Serena Toffanin, Cristiana Bulato, Elena Campello, Paolo Simioni, Luca Spiezia

**Affiliations:** General Internal Medicine & Thrombotic and Haemorrhagic Diseases Unit, Department of Medicine, Padova University School of Medicine, Padova, Italy

**Keywords:** transthyretin amyloidosis, thrombotic complications, thromboelastometry thrombin generation, extracellular vesicles, case report

## Abstract

Cardiac amyloidosis is a group of diseases characterized by the deposition of amyloid fibers in cardiac tissue. Two forms are mainly reported: light chain (AL) and transthyretin (ATTR) amyloidosis. Among the complications of transthyretin amyloidosis there are thrombotic events and, to a lesser extent, hemorrhagic events. The latter are likely caused by perivascular amyloid deposition resulting in capillary fragility, in addition to INR lability during anticoagulant therapy. The onset of thrombotic events may be caused by the high prevalence of atrial fibrillation (AF), mechanical cardiac dysfunction and atrial myopathy observed in patients with transthyretin amyloidosis. It remains unclear why thromboembolic events occur even in patients with sinus rhythm or adequate anticoagulation, though a hypercoagulable state or underlying inflammation may be involved. We report a case of cryptogenic ischemic stroke in an 86-year-old woman with transthyretin amyloidosis and sinus rhythm. Traditional coagulation tests, whole blood rotational thromboelastometry and impedance aggregometry did not show a hypercoagulable state. The thrombin generation assay did not reveal a prothrombotic state. However, the study of extracellular vesicles highlighted underlying immune-mediated endothelial damage likely responsible for the thrombotic diathesis. It could be hypothesized that inflammation plays a role in the hypercoagulability of patients with transthyretin amyloidosis. Larger prospective studies are needed to validate our hypothesis.

## Introduction

Cardiac amyloidosis is a group of diseases caused by the deposition of amyloid fibers in heart tissue. There are two main forms of this disease: immunoglobulin light chain amyloidosis (AL); and transthyretin amyloidosis (ATTR) which is subdivided into hereditary transthyretin (TTR) amyloidosis (ATTRv) and wild-type TTR amyloidosis (ATTRwt) (1, 2). Among the most important complications of ATTR are thrombotic events and less frequently, hemorrhagic episodes (3). Although the latter appear to stem from perivascular amyloid deposition and consequent capillary fragility as well as labile INR during anticoagulant therapy, little is known about the mechanisms underlying thromboembolic events in patients with ATTR (4). The most common thrombotic events in ATTR amyloidosis are intracardiac thrombi and cerebral ischemic events, and less frequently, peripheral and splanchnic thrombosis. Amyloidosis fibers infiltrate the atrium and cause mechanical dysfunction resulting in blood stasis as well as endothelial damage, both elements of Virchow's triad that promote thrombosis. Hence the high prevalence of atrial fibrillation (AF), mechanical cardiac dysfunction and atrial myopathy in patients with ATTR. It remains unclear why thrombosis may occur even in patients with sinus rhythm and, albeit rarely, in adequately anticoagulated patients (5). Transthyretin may also be involved in the activation and regulation of the coagulation and fibrinolytic systems (6), which may contribute to the onset of thrombosis. We report a case of cryptogenic ischemic stroke in an 86-year-old female patient with transthyretin amyloidosis and sinus rhythm who underwent extensive coagulation, vascular and inflammatory workups to identify the possible pathogenetic mechanisms underlying the thrombotic event.

## Methods

After obtaining written informed consent and following overnight fasting, blood samples were collected 37 days after the acute event, by venipuncture directly into 5 BD Vacutainer® Citrate Tubes containing 0.109 M (3.2%) sodium citrate (9:1 blood to anticoagulant ratio) using a 21 gauge needle with light tourniquet, after discharging the first mL of venous blood. Platelet-poor plasma (PPP) was prepared within 1 h of blood collection by double centrifugation at 2,500 × g for 15 min at room temperature. Aliquots (0.5 mL) were immediately frozen and stored at −80 °C until analysis.

### Traditional coagulation tests

We measured prothrombin time/international normalized ratio, activated partial thromboplastin time, FXI, FX, FIX, FVIII, FII, fibrinogen, antithrombin (AT), protein C (PC), and protein S (PS), plasminogen, plasminogen activator inhibitor-1 (PAI-1) antigen, alpha2-antiplasmin, lupus anticoagulant (LA), anticardiolipin (aCL) antibodies (IgG and IgM), and anti-beta-2 glycoprotein 1 (anti-β2GP1) antibodies (IgG and IgM). Moreover, the presence of FV Leiden mutation and prothrombin G20210A variant was assessed. Prothrombin time (PT, n.v. 70%–100%), international normalized ratio (INR) and activated partial thromboplastin time (aPTT, n.v. 22.8–31 s) were measured according to standard procedures. Activity levels of FXI (n.v. 80%–120%), FX (n.v. 80%–120%), FIX (n.v. 80%–120%), FVIII (n.v. 60%–160%), and FII (n.v. 80%–120%) were measured using specific factor-deficient plasma (Siemens Healthcare Diagnostics, Milan, Italy). Fibrinogen concentration (n.v. 150–450 mg/dl) was measured by the Clauss method using a BCT-Analyzer (Dade Behring, Marburg, Germany) according to the manufacturer's recommendations. AT activity (n.v. 80%–120%) was measured using a thrombin-based chromogenic substrate assay (Berichrom® Antithrombin III, Siemens Healthcare Diagnostics) and PC activity (n.v. 80%–120%) was measured using a commercial kit (Protein C Reagent, Siemens Healthcare Diagnostics, Milan, Italy). AT and PC were performed using a BCS XP coagulation analyser (Siemens Healthcare Diagnostics, Milan, Italy). PS activity (n.v. 70%–130%) was assessed using the ProS kit (Instrumentation Laboratory, Milan, Italy) on an ACL TOP 300 CTS coagulation analyser (Instrumentation Laboratory, Milan, Italy). Coagulation inhibitors were measured according to the standard protocols supplied by the manufacturer, as previously reported (7). Plasminogen (n.v. 75%–140%), PAI-1 antigen (1.0–25.0 ng/ml) and alpha2-antiplasmin (n.v. 80%–120%) were measured according to standard's procedures. LA (dRVVT, n.v. 26–45 s and aPTT-LA, n.v. 32–43 s), aCL antibodies (IgG and IgM) (n.v. < 8 U/ml, in both cases) and anti-β2GP1 antibodies (IgG and IgM) (n.v. < 10 U/ml, in both cases) were measured as previously reported (8). Finally, genetic polymorphisms were determined using ABI Prism 3100 Genetic Analyzer (Applied Biosystem, Waltham, MA, USA) according to the manufacturer's instructions.

### Whole blood coagulation tests

Rotational thromboelastometry (ROTEM® apparatus, Werfen, Bedford, MA, USA) is a whole blood (WB) viscoelastic test that evaluates changes in the viscoelastic resistance of the blood by applying a constant rotational force, and globally assesses the coagulation process from clot formation and stabilization to lysis (9). We performed the ROTEM® analysis according to the standard protocols supplied by the manufacturer, as previously reported (10, 11). The following tests were performed: INTEM (to assess intrinsic coagulation pathway), EXTEM (to assess the extrinsic coagulation pathway) and FIBTEM (to assess fibrinogen contribution to clot formation and stability). For EXTEM and INTEM assays, the following ROTEM® parameters were considered: (*i*) clotting time (CT, sec), the time from the beginning of the coagulation analysis until an increase in amplitude of 2 mm; (*ii)* clot formation time (CFT, sec), the time between an increase in amplitude of the thromboelastogram from 2 to 20 mm; (*iii)* maximum clot firmness (MCF, mm), the maximum amplitude reached in the thromboelastogram; (*iv)* alpha-angle (*α*, degrees), the angle between the horizontal axis and tangential line to the thromboelastogram through the 2 mm amplitude; (*v)* maximum lysis (ML, %), a measure of the percent of decrease in amplitude of the thromboelastogram at the end of the test. For FIBTEM assay CT, MCF and ML were considered. The CT reflects the initiation phase of the clotting process, CFT and alpha angles describe clot kinetics, MCF reflects clot stiffness and ML indicates the speed of fibrinolysis.

Platelet aggregation was tested using the Multiplate® function analyzer (Roche Diagnostics GmbH, Mannheim, Germany). Impedance aggregometry is a platelet function test in WB used to diagnose platelet alterations, in the monitoring of antiplatelet therapy and as a potential predictor of the need for transfusion and bleeding risk during major surgical procedures. The instrument continuously measures the changes in the electrical resistance (called “impedance”) between two copper wires. The greater the area, the more platelets aggregate. Briefly, 300 μl of citrated WB were added to an equal amount of saline solution, preheated at 37 °C, and platelet aggregation was tested after specific activation with arachidonic acid (ASPItest), adenosine diphosphate (ADPtest) and a thrombin analogue (TRAPtest). Platelet aggregation was electronically measured for 6 min and expressed as units of area under the curve (AUC) plotted over time in arbitrary units (U) (1 U = 10 AU × min) (12).

Thrombin generation (TG) was assessed using the calibrated automated thrombogram method (Thrombinoscope BV, Diagnostica Stago, Maastricht, The Netherlands) according to manufacturer's instructions (13). The test was performed on citrated WB within 4 h of collection, with and without the addition of thrombomodulin 20 nM (TM; Synapse Research Institute, Maastricht, the Netherlands) as previously described (14, 15). Citrated WB was firstly mixed with the fluorogenic thrombin substrate Z-Gly-Gly-Arg-aminomethylcoumarin (ZGGR-AMC; Bachem, Basel, Switzerland) and incubated for 10 min at 37 °C. Subsequently, WB was mixed with a solution containing tissue factor (TF) (Innovin®, Siemens Healthcare Diagnostics, Marburg, Germany) CaCl_2_ and MgCl_2_ in the presence or absence of TM. The volume ratio of WB, substrate solution, and TF-containing solution was 3:1:2. Sixty-five μl of the resulting mixture was transferred into wells of a 96-well plate. The final concentrations in the well were 50% WB, 1 pmol/L TF, 6 mmol/L CaCl_2_, 3 mM MgCl_2_, and 416.7 μmol/L ZGGR-AMC in the absence or presence of 20 nmol/L TM. The concentration of TM was chosen based on dose-response experiments in healthy controls, to inhibit TG by approximately 50%. All blood samples were measured in triplicate and calibrated by replacing the TF-containing solution with *α*_2_-macroglobulin-thrombin complex (*α*_2_M-T, corresponding with 300 nmol/L thrombin activity). Fluorescence signals were recorded at 37 °C with an interval time of 6 s by a microplate fluorometer (Fluoroskan AscentTM, Thermo Labsystems, Helsinki, Finland) with *λ*_ex_ = 485 nm and *λ*_em_ = 538 nm using Fluoroskan Ascent Software (version 2.6). TG curves were described in terms of endogenous thrombin potential (ETP, nM*min), peak (nM), lag time (min), time to peak (min) and velocity index (vel index, nM/min). The results obtained were compared to those of a small (n. 20) historical group of healthy subjects previously described (16).

### Extracellular vesicles

The pre-analytic phase of Extracellular vesicles (EV) analysis has been reported previously (17). We thawed PPP in a water bath for 5 min at 37 °C and after isolating large extracellular vesicles (L-EVs) by centrifugation at 14,000 g for 30 min at 4 °C, samples were immediately processed for immunolabeling. Samples were analyzed after a single freeze-thaw cycle. Flow cytometry analysis was performed using a CytoFLEX S flow cytometer (Beckman Coulter, United States), as previously reported (17, 18). For EV size calibration of the flow cytometer, fluorescent polystyrene beads Gigamix a mix 1:1 of Megamix FSC & SSC Plus (BioCytex, Marseille, France) were used in sizes of 0.1, 0.16, 0.2, 0.24, 0.3, 0.5, and 0.9 μm. Violet side scatter (VSSC) and FL1 channel gain were set to visualize the beads. The side scatter (SSC) from the 405 nm violet laser (VSSC) was used as a trigger signal to discriminate the noise with a size detection limit of 80 nm. Gigamix bead solution was gated to exclude background noise (from the solution itself). After turning the set in VSSC and forward scatter (FSC), a rectangular gate was set between the 0.2 and 0.9 μm bead populations and defined as L-EVs gate. Prior to staining, the antibody mixtures were centrifuged at 20,000 g for 30 min to remove fluorescent particles. The final concentration of the antibodies used was 1–5 μg/ml. The dilution buffer used, PBS and Annexin V buffer (Abcam, ab214484), were sterilized through a 0.2 µm mesh filter to reduce background noise. L-EVs were labeled with calcein-AM (referred to calcein-green, EMD Millipore Corp., cod.CI206700-1MG), Alexa Fluor 647-labeled Annexin V (Abcam, cod.ab214484), phycoerythrin (PE)-labeled anti-CD62E antibody (clone HAE-1f; BioLegend, San Diego, CA, cod.336008), mouse anti-human-TF (1:100 diluted, #4509, American Diagnostica) followed by anti-mouse IgG secondary antibody Alexa Fluor 750 (1:300 diluted, Sigma-Aldrich, cod.SAB4600211-250UL), PE-labeled anti-CD62P (clone CLB-Thromb/6; Beckman Coulter, Marseille, France, cod.IM1759U) PE/Cyanine 7-labeled anti-CD41 (clone P2; Beckman Coulter, cod.6607115), ECD labeled anti-CD45 (clone J33; Beckman Coulter, cod.A07784), PE-labeled anti-CD14 (clone 61D3; Invitrogen, cod.12-0149-42). Calcein-AM staining and the non-conjugated primary antibodies were incubated for 30 min at 37 °C. Subsequently, the conjugated antibodies and the secondary antibodies (i.e., anti-mouse IgG Alexa Fluor 750), were incubated for 30 min in the dark at RT. Finally, L-EVs were resuspended in 120 μl of sterile filtered Annexin V buffer using a 96-well polypropylene flat plate and analyzed within 1 h of immunolabeling. Parallel incubation was performed with isotype-matched control antibodies and with the secondary antibody alone to exclude non-specific staining. Fluorescence measured with the respective isotype negative control antibody were subtracted in order to avoid non-specific background signal. Unstained samples were used as negative control. Flow cytometer acquisition settings were maintained for all samples, including triggering threshold, voltages and flow rate. No substantial changes in scatter or fluorescence signals were observed between unstained and matched isotype controls. We expressed EVs as events/*μ*l (absolute count) with the volume measurement of the CytoFLEX. Files were exported and data was evaluated by CytExpert (Software Version 2.3, Beckman Coulter). True EV events were defined as double-positive stained for calcein-AM and one of the other specific antibodies. Triple-positive EVs were also evaluated for calcein-AM, anti-CD62E and anti-TF, and namely, E-Selectin^+^TF^+^. Calcein-AM is a non-fluorescent marker for EVs which becomes fluorescent only after absorption due to the esterase present in the double phospholipid membrane of EVs (calcein^+^). Annexin V is an index of apoptosis which binds to phosphatidylserine, a membrane phospholipid whose presence on the external surface after activation of platelets promotes coagulation and thrombosis. CD62E/E-Selectin^+^ is a marker of activated endothelial cells; CD62P/P-Selectin^+^, marker of activated platelets; CD41, marker of platelet-derived EVs; CD45, marker of leukocyte-derived EVs and CD14, marker of monocyte/macrophage-derived EVs. The levels of EVs in our patient were compared to those observed in 20 healthy age- (±2 years) and sex-matched controls.

## Case report

**Figure 1:**
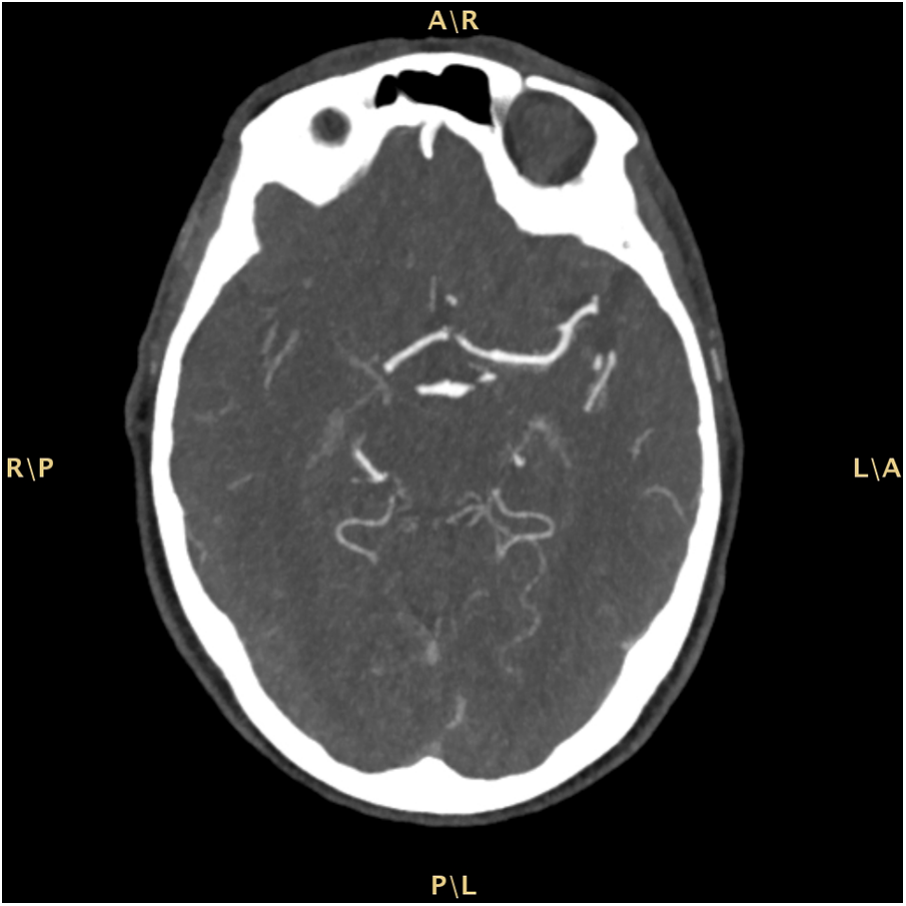
Computed tomography (CT) scan of the brain.

The patient was admitted to our Emergency Department (ED) due to a sudden onset of left hemisyndrome and deviation of gaze to the right, as witnessed by family members. Past medical history included a previous tuberculosis with right-sided cavitation and transthyretin cardiac amyloidosis diagnosed in May 2022 — i.e., suggestive echocardiographic findings with left ventricular hypertrophy without cardiac kinetic abnormalities; absence of monoclonal serum component in blood chemistry and urinary tests; supported by a bone scintigraphy with cardiac hyperuptake (Perugini score 3). Moreover, the patient was undergoing antihypertensive and anxiolytic therapies. White blood cell count and hemoglobin were within the normal ranges. Renal and liver function tests were normal. Pre-stroke mRS score was 1. A computed tomography (CT) scan of the brain showed a malacic area limited to the left mesial frontal region, traceable to an ischemic event in the region of the distal branches of the ipsilateral anterior cerebral artery (Alberta stroke program early CT score, ASPECTS 9). A CT angiography of the cerebral-afferent vessels combined with a perfusion study showed no recognizable opacification in the M1 section of the right middle cerebral artery, due to thrombotic occlusion. The perfusion study revealed abnormal Tmax and cerebral blood flow (CBF) values in the volume of parenchyma studied with a mismatch of approximately 21.3, due to the presence of a large area of ischemic penumbra in the region of the right middle cerebral artery (Figure 1). The neurological exam performed in the ED showed a lack of leveling of the left upper limb and sagging of the left lower limb, and fluent speech albeit with dysarthria (National Institutes of Health Stroke Scale, NIHSS 12). Since there were no contraindications, systemic thrombolysis was performed for a weight of 73 kg, total dose 65.7 mg (10% bolus, 1 hour of continuous infusion). The patient was later transferred to the angiography suite to undergo mechanical thrombectomy, with manual thromboaspiration of the organized red thrombus. The final control angiography showed complete recanalization of the right middle cerebral artery (modified thrombolysis in cerebral infarction, mTICI 3). Finally, the patient was admitted to the Stroke Unit. During hospitalization the patient remained haemodynamically stable, with good respiratory exchange and afebrile. The electrocardiogram (ECG) showed a first-degree atrioventricular block, right bundle branch block and left anterior fascicular block. The patient was under continuous electrocardiography monitoring throughout her stay (4 days) in the Stroke Unit after the acute event, and no atrial fibrillation was detected. Vital parameters remained normal and stable during hospitalization. The control brain CT scan performed on the first day of hospitalization confirmed an ischemic lesion in the right internal capsule. A neurosonological study of the intracranial and cerebral-afferent vessels to ascertain its etiology, confirmed the patency of the right middle cerebral artery whereas there was a significant stenosis before the procedure. Furthermore, a resting echocardiography also revealed only moderate left atrial dilatation and mild mitral regurgitation. Finally, a 24-h Holter monitor detected no occult arrhythmias. A full cardiological evaluation recommended continuation of the ongoing follow-up for cardiac amyloidosis. Acetylsalicylic acid was initiated as secondary prevention therapy and atorvastatin was also introduced at low dosage due to the vascular pleiotropic effect. A Doppler ultrasound of the supra-aortic trunks revealed an extracranial finding of mild stenosis of the carotid bifurcation and at the origin of the right external carotid artery and the Doppler ultrasound of the intracranial vessels instead was within normal limits. The patient underwent cycles of kinesiotherapy with good response. The discharge neurological exam showed a clear improvement: a slight weakness of the left lower extremity and a slight impairment of the left seventh cranial nerve persisted. The patient walked unassisted with the aid of a walker and was fed orally with a soft diet and free fluids.

**Figure 2:**
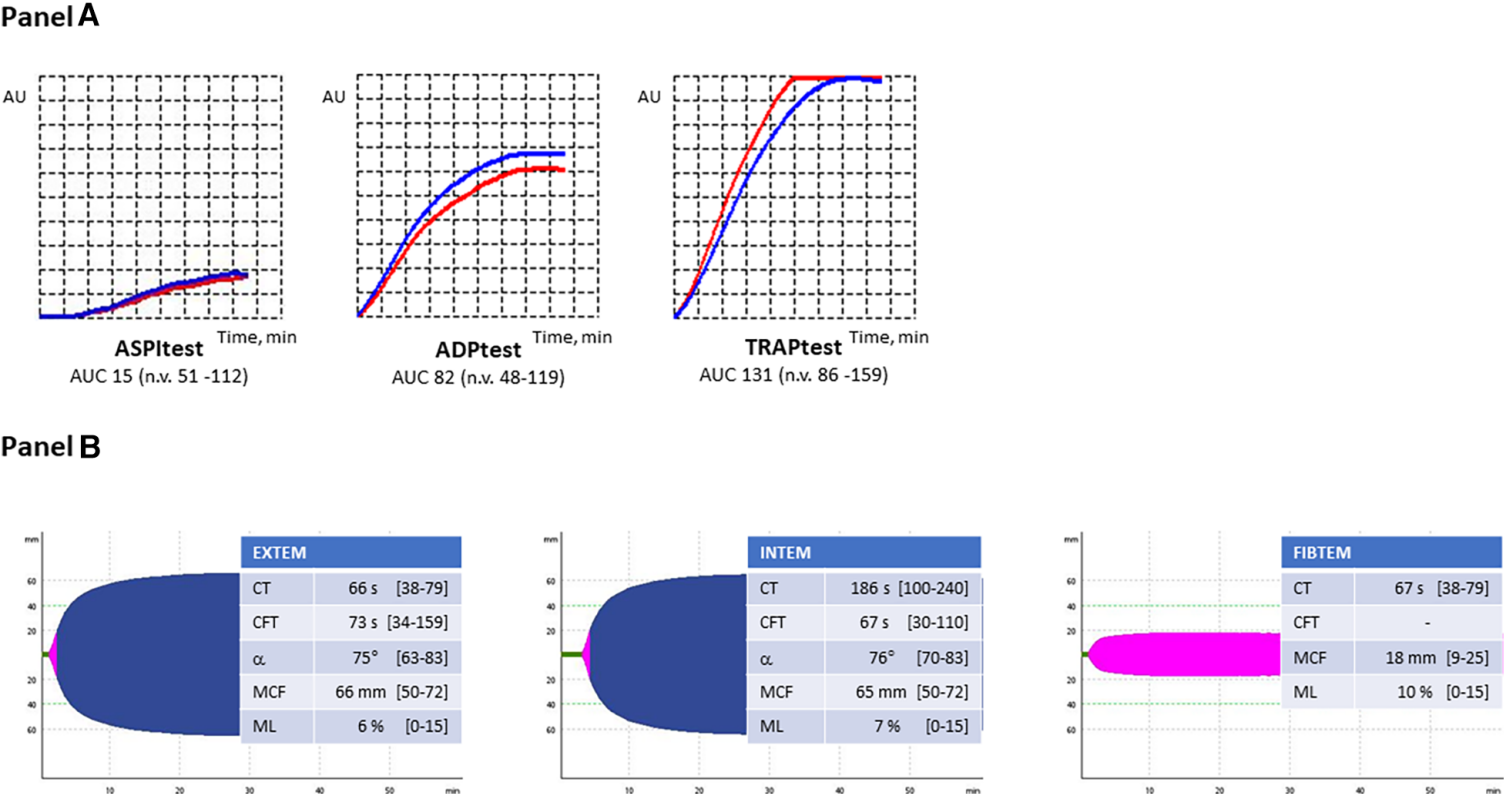
(**A**) Multiplate aggregometry profiles. ASPI-test, arachidonic acid-induced aggregation; ADP-test, adenosine-diphosphate-induced aggregation; TRAP-test, thrombin receptor activating peptide-induced aggregation. (**B**) Thromboelastometry profiles. EXTEM, extrinsic coagulation pathway assay; INTEM, intrinsic coagulation pathway assay; FIBTEM, contribution of fibrinogen to blood clotting assay; CT, clotting time; CFT, clot formation time; ɑ, ɑ-angle; MCF, maximum clot firmness; ML, maximum lysis.

The coagulation parameters of our patient are reported in Table 1, and they were all normal. The search for aCL antibodies and anti-β2GP1 antibodies was negative. Genetic testing excluded the presence of the FV Leiden mutation and prothrombin G20210A variant. The thromboelastometry profiles and Multiplate® aggregometry results are reported in Figure 2. All parameters considered were within the normal ranges, except the AUC value in ASPItest which was significantly reduced vs. the lower limit of the normal range, likely due to ongoing antiplatelet therapy at the time of blood sample collection. The results of WB-TG are reported in Table 1. The values observed were within the range (25th-75th percentile) obtained in a small cohort (n. 10) of healthy subjects. Our patient's L-EVs measurements are reported in Table 2. We found that endothelium-derived (CD14^+^ and E-selectin) and leukocyte-derived (CD45^+^) L-EVs, as well as E-selectin^+^TF^+^ L-EVs were higher vs. healthy controls. We also observed an increase in the absolute count of L-EVs vs. healthy controls. Conversely, platelet-derived L-EVs (CD41^+^, P-selectin and CD41^+^P-selectin^+^) were lower in our patient vs. healthy controls.

**Table 1:** Traditional coagulation tests and thrombin generation.

	Value	Reference range
Prothrombin time, PT (%)	80.4	70–100
International normalized ratio, INR	1.11	–
Activated partial thromboplastin time, aPTT (sec)	30.7	22.8–31.0
FXI (%)	80.6	80–120
FX (%)	83.6	80–120
FIX (%)	88.2	80–120
FVIII (%)	119.6	60–120
FII (%)	84.9	80–120
Fibrinogen (mg/dl)	238	150–450
Antithrombin (%)	83.3	80–120
Protein C coagulometric activity (%)	80.9	80–120
Protein C chromogenic activity (%)	85.7	80–120
Protein C antigen (%)	84	80–120
Protein S coagulometric activity (%)	81.6	70–130
Protein S free antigen (%)	102	80–120
Protein S total antigen (%)	91	80–120
Plasminogen activity (%)	73.6	75–140
Plasminogen activator inhibitor-1 antigen (ng/ml)	11.6	1.0–25.0
Alpha2-antiplasmin (%)	80.9	80–120
aPTT—Lupus Anticoagulant (sec)	33.7	32.0–43.0
dRVVT (sec)	32.8	26.0–45.0
Anticardiolipin antibodies IgG (U/ml)	1.2	<10
Anticardiolipin antibodies IgM (U/ml)	2.3	<10
Anti-beta-2 glycoprotein 1 IgG (U/ml)	1.4	<8
Anti-beta-2 glycoprotein 1 IgM (U/ml)	2.0	<8
APC sensitivity ratio	3.71	>2.00
APC sensitivity ratio normalization	1.23	>0.84
FV Leiden mutation	Wild type	–
Prothrombin G20210A variant	Wild type	–
Thrombin Generation		
ETP (nM*min)	1,525	1,374–1,804
ETP + TM (nM*min)	893	755–1,138
Peak (nM)	182.9	175.6–228.9
Peak + TM (nM)	119.9	109.6–132.7
Lag time (min)	3.7	3.2–4.3
Lag time + TM (min)	5.7	5.1–6.5
Time to peak (min)	8.8	8.3–10.0
Time to peak + TM (min)	9.9	9.5–11.5
Vel index (nM/min)	38.0	29.3–39.7
Vel index + TM (nM/min)	28.2	24.5–37.6

dRVVT, diluted Russel viper venom time; APC, activated protein C; ETP, endogenous thrombin potential; TM, thrombomodulin, Vel, velocity.

**Table 2:** Large EV subgroups levels.

	Patient	Controls
Calcein ^+ ^Annexin V^+^	113	328
Calcein ^+ ^CD41^+^	492	1,052
Calcein ^+ ^P-selectin^+^	614	2,216
Calcein ^+ ^CD41 ^+ ^P-selectin^+^	41	259
Calcein ^+ ^CD45^+^	1,443	1,673
Calcein ^+ ^CD14^+^	102	73
Calcein ^+ ^E-selectin	1,540	1,261
Calcein ^+ ^TF^+^	107	79
Calcein ^+ ^E-selectin ^+ ^TF^+^	56	31

Data shown as median in events/µl with the volume measurement of the.

CytoFLEX (Beckman Coulter). EV, extracellular vesicle; TF, tissue factor.

## Discussion

Thrombotic events are among the most severe complications of amyloidosis, particularly in ATTR and mainly as it pertains to intracardiac thrombi and cerebrovascular events. In a recently published review (19), we endeavored to identify studies that investigated the incidence of and the risk factors associated with thromboembolism in patients with ATTR — there were few and comprising small cohorts. The study by Bukari S et al. (20) confirmed a high thromboembolic risk in patients with ATTRwt-CA and AF, considering the latter was present on average in 88% of patients. It has been noted that even ATTR patients in sinus rhythm or in atrial fibrillation with adequate anticoagulant therapy may develop both atrial and cerebrovascular thromboembolic events, as in our case, and more rarely, peripheral thrombosis. In fact, a study by Vilches et al. (21) found a 1.3 incidence rate among subjects with normal sinus rhythm without oral anticoagulant therapy (OAT), 1.7 among those with AF undergoing OAT, and 4.8 among those with AF without OAT. It is of the utmost importance to understand the mechanisms underlying thromboembolism in these patients, particularly whether the coagulation system is involved, to identify who might benefit from early anticoagulant treatment. Thus, our patient underwent an extensive coagulation workup (i.e., the extrinsic and intrinsic coagulation pathways, fibrinolysis, antiphospholipid antibody syndrome and inherited thrombophilia testing). All these tests excluded the presence of a prothrombotic state. Moreover, those tests performed in WB, and in particular thromboelastometry, impedance aggregometry and TG all failed to detect a hypercoagulable state which may have explained the thrombotic episode suffered by our patient. It bears noting that the ASPI test highlighted a markedly reduced platelet function, traceable to the fact that our patient was taking antiplatelet drugs at the time of blood sampling. Finally, we performed an analysis of EVs which are cell-derived vesicles delimited by a phospholipid bilayer, with a diameter of 30–1,000 nm, released by extrusion of the cytoplasmic membrane of activated cells in normal (physiological) conditions and during diseases (pathological conditions), and considered as mediators of intercellular communication (22). There have been reports that L-EVs may have procoagulant properties due to the presence of negatively charged membrane phospholipids (i.e., phosphatidylserine) on their surface, which provide a substrate for the protagonists of coagulation (intrinsic pathway) but can also trigger coagulation (extrinsic pathway) through the expression of tissue factor (23). The origin of L-EVs can be identified by studying surface antigens (e.g., activated endothelial cells, platelets, leukocytes) and the different subtypes can be quantified (24). Our main endeavor was to measure EV levels once the patients had recovered or overcome the active phase of the stroke, to ascertain whether there was any correlation between “baseline” EV levels and cardiac amyloidosis. In our case, it had been over a month since the acute event (37 days) which placed our patient in the early subacute phase according to the traditional classification of ischemic stroke. Furthermore, the EV trend according to the study by Lundström et al. (25) would place us towards the end of the convalescence phase, hence the reason why we deemed this time window appropriate for blood sampling. We compared our patient's EV profile to that observed in 20 healthy age- and sex-matched controls. Interestingly, both endothelium-derived and leukocyte-derived L-EVs measured in our patient were higher vs. control group. We hypothesized that these findings may suggest an underlying immune-mediated endothelial damage responsible for the thrombotic diathesis, and therefore, inflammation may contribute to the coagulopathy in patients with ATTR. To the best of our knowledge there are no other studies published so far in the literature that have evaluated the circulating levels of L-EVs in patients with ATTR. However, it bears noting that lower levels of platelet-derived EVs in our patient vs. controls is not an unexpected finding. There have been reports in the literature that antiplatelet drugs, as effectively administered in our patient given the ASPItest results, may reduce plasma levels of platelet-derived L-EVs (26). Bulut D et al. (27) demonstrated a reduction in the number of circulating L-EVs derived from endothelium and platelets in 15 male patients with coronary artery disease being treated with aspirin. Another interesting case was reported by Kim HJ et al. (28) wherein after the discontinuation of aspirin in 17/46 patients presenting with a chronic inflammatory disease such as Kawasaki syndrome, there was a renewed increase in circulating platelet-derived L-EVs. The hypothesis is that there may be an inflammatory substrate in ATTR amyloidosis as well. The study by Lv Y at al (29). also suggests that treatment with aspirin can reduce the number of circulating endothelial cells and platelet-derived L-EVs in patients with cardiovascular diseases. On the other hand, we would argue that ascertaining the effect of statins on endothelium-derived EVs is still a matter of debate, and it has been evaluated mostly in cultured endothelial cells (30). Statins have been shown to reduce levels of circulating EVs derived from endothelium, platelets and inflammatory cells (31). Although there are several studies in the literature demonstrating that statins downregulate the release of endothelium-derived EVs by human coronary artery endothelial cells (32–34), few studies have investigated the effects of statins on platelet function. These studies demonstrate that there are pathways potentially responsible for interactions between statins and platelets, and support the hypothesis that statin treatment may reduce not only the number of platelet-derived EVs but markers of activated platelets as well (35).

We would be remiss not to mention some of the limitations of our study. We did not perform a high-resolution brain Magnetic Resonance Imaging (MRI) and thus were not able to exclude the presence of cerebral small vessel disease—such as, cerebral amyloid angiopathy (CAA)—as a cause of ischemia. Nevertheless, our patient did not present a clinical history compatible with CAA and the acute event occurred in a large vessel (i.e., the right middle cerebral artery, on cerebral CT angiography) requiring thrombolysis, without hemorrhagic complications, as seen on subsequent CT scans. Therefore, we deemed the hypothesis of CAA unlikely. Moreover, we could not definitively exclude a cardioembolic etiology for the cerebral ischemic event in our patient. Throughout hospitalization, we found no evidence of atrial fibrillation and the echocardiogram revealed no intracardiac thrombi. Nevertheless, we cannot definitively rule it out. The use of fluorescent polystyrene beads for EV size calibration constitutes another limitation as they do not have the same refractive index as EVs, which may also vary by size. Another limitation of the study, according to the latest minimal information for studies of extracellular vesicles (MISEV) recommendations, is that no single measurement or method is able to satisfy all EV characterization requirements, and the use of different orthogonal techniques is recommended. Unfortunately, we did not perform electron microscopy or nanoparticle tracking analysis (NTA) as these techniques require large volumes of plasma sample.

In conclusion, our findings are preliminary and did not allow us to draw any definitive conclusions on the possible use of L-EVs as predictive biomarkers in clinical practice. Larger prospective studies are needed to confirm our findings and to clarify the role, if any, of L-EVs as a possible risk factor of thrombotic events in patients with ATTR.

## Data Availability

The original contributions presented in the study are included in the article/Supplementary Material, further inquiries can be directed to the corresponding author.
